# Pediatric Extraspinal Sacrococcygeal Ependymoma: Report of Two Cases and Literature Review

**DOI:** 10.3390/diagnostics11091680

**Published:** 2021-09-15

**Authors:** Francesco Fabozzi, Silvia Ceccanti, Antonella Cacchione, Giovanna Stefania Colafati, Andrea Carai, Alessandro Crocoli, Angela Mastronuzzi, Denis A. Cozzi

**Affiliations:** 1 Department of Hematology/Oncology, Cell and Gene Therapy, IRCCS Bambino Gesù Children’s Hospital, 00165 Rome, Italy; francesco.fabozzi@opbg.net (F.F.); antonella.cacchione@opbg.net (A.C.); 2 Pediatric Surgery Unit, Sapienza University of Rome, AOU Policlinico Umberto I, 00161 Rome, Italy; silvia.ceccanti@uniroma1.it (S.C.); da.cozzi@uniroma1.it (D.A.C.); 3 Neuroradiology Unit, Department of Imaging, IRCCS Bambino Gesù Children’s Hospital, 00165 Rome, Italy; gstefania.colafati@opbg.net; 4 Neurosurgery Unit, Department of Neurosciences, IRCCS Bambino Gesù Children’s Hospital, 00165 Rome, Italy; andrea.carai@opbg.net; 5 Department of Pediatric Surgery, IRCCS Bambino Gesù Children’s Hospital, 00165 Rome, Italy; alessandro.crocoli@opbg.net

**Keywords:** pediatric ependymoma, extra-CNS ependymoma, myxopapillary ependymoma, pilonidal disease

## Abstract

Primary central nervous system (CNS) tumors represent the most common solid tumors in childhood. Ependymomas arise from ependymal cells lining the wall of ventricles or central canal of spinal cord and their occurrence outside the CNS is extremely rare, published in the literature as case reports or small case series. We present two cases of extra-CNS myxopapillary ependymomas treated at our institution in the past three years; both cases originate in the sacrococcygeal region and were initially misdiagnosed as epidermoid cyst and germ cell tumor, respectively. The first case, which arose in a 9-year-old girl, was treated with a surgical excision in two stages, due to the non-radical manner of the first operation; no recurrence was observed after two years of follow-up. The other case was a 12-year-old boy who was treated with a complete resection and showed no evidence of recurrence at one-year follow-up. In this paper, we report our experience in treating an extremely rare disease that lacks a standardized approach to diagnosis, treatment and follow-up; in addition, we perform a literature review of the past 35 years.

## 1. Introduction

Ependymoma comprises approximately 9% of all pediatric central nervous system (CNS) tumors, representing the most common spinal cord neoplasm of this age group [1]. It originates from ependymal cells that line the wall of ventricles or central canal of spinal cord. According to the upcoming 2021 World Health Organization (WHO) classification of CNS tumors, ependymal tumors are now classified into the following subtypes, based on anatomic site and a combination of histopathological and molecular features [2]: supratentorial ependymoma; supratentorial ependymoma, *ZFTA* fusion-positive; supratentorial ependymoma, *YAP1* fusion-positive; posterior fossa ependymoma; posterior fossa ependymoma, group PFA; posterior fossa ependymoma, group PFB; spinal ependymoma; spinal ependymoma, *MYCN*-amplified; myxopapillary ependymoma; subependymoma. In particular, myxopapillary ependymoma is now considered CNS WHO grade 2 instead of 1, since its likelihood of recurrence is currently known to be similar to that of conventional spinal ependymoma.

Among all pediatric brain and spinal tumors, ependymoma occasionally arises outside the CNS. Various localizations are described in the literature: the most common during childhood is the subcutaneous tissue of the sacrococcygeal region, whereas less frequently is the presacral region; rarer localizations can include the mediastinum, liver, or lung, described mainly in adulthood [3,4,5]. Here, we report two cases of ependymoma of the sacrococcygeal region treated at our institution, with a review of the literature regarding this rare tumor in children.

## 2. Case Presentation

### 2.1. Case 1 

A 9-year-old girl presented at an outside institution with a history of a persistent mass in the gluteal region; no associated symptoms were reported. The presence of a congenital sacral dimple was reported in the anamnesis. She underwent lumbosacral magnetic resonance (MRI) which revealed the presence of T2 hyperintense capsular formation measuring 30 × 15 mm in the soft tissues of the left paramedian posterior sacrococcygeal region, without any intrinsic spinal cord component nor connection to the central canal. An epidermoid cyst was hypothesized, and the girl was then subjected to surgical removal of the lesion. Surprisingly, histological examination revealed a moderately cellular neoplasm with areas of fibromyxoid stroma. An immunohistochemical analysis showed the neoplastic cells were strongly positive for glial fibrillary acidic protein (GFAP) and S-100, whereas they were negative for cytokeratin (AE1/AE3), epithelial membrane antigen (EMA) and Olig2. These features allowed for the diagnosis of myxopapillary ependymoma. Proliferation index evaluated with Ki-67 was 2–3%, reaching values of 9–10% in the central part of the neoplasm. The surgical margins were positive, showing the non-radicality of the resection. Due to the rarity of the neoplasm, the girl was then referred to our center. Because myxopapillary ependymoma has the potential for metastatic spread [6].,the tumor was staged using a total body computer tomography (CT), resulting negative for systemic localizations. Local re-evaluation, carried out with full neuraxis MRI, revealed an inhomogeneous signal of the surgical area, although no images referable to disease recurrence or secondary localization were found. Considering the positive surgical margins, the girl underwent reoperation for extension of the surgical margins and coccygectomy. Histological examination confirmed the absence of local recurrence. Brain and spine MRI follow-up was established every four months during the first year and every six months thereafter, confirming complete remission two years after surgery.

### 2.2. Case 2 

**Figure 1:**
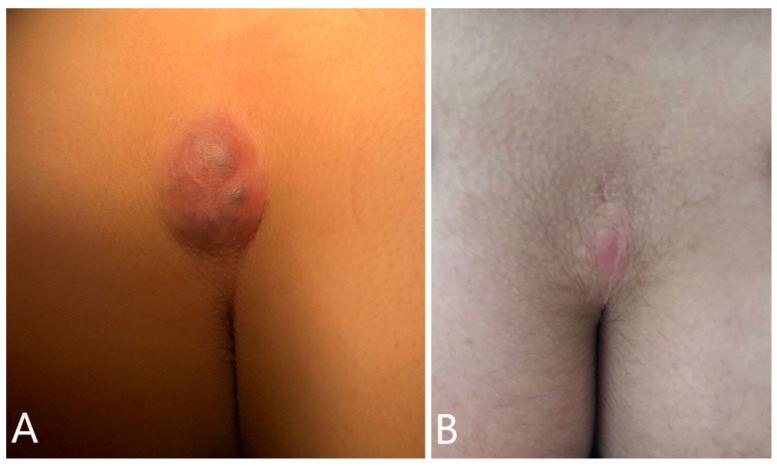
Visible subcutaneous sacrococcygeal swelling, firmly fixed to overlying discolored intact skin, and loosely adherent to the coccyx (**A**). Postoperative appearance of the sacrococcygeal region 1 year after surgery, showing a well-healed scar and normal skin color (**B**).

A 13-year-old boy with a history of a congenital slow-growing sacrococcygeal swelling, sought care for an increase in volume and one episode of self-limiting inflammation of the lesion in the last two months (Figure 1A). Ultrasonography showed a solid ovoid formation with an inhomogeneous echo structure, measuring 39 × 31 × 19.5 mm, adhering to the posterior profile of the sacrum, without evidence of sinus tracts. Due to suspicion of germ cell tumor, serum beta-HCG and alpha-fetoprotein levels were requested, resulting within normal limits. He subsequently underwent surgical resection of the lesion, including the overlying skin. The histological examination showed a neoplastic lesion characterized by several microcystic-hemorrhagic areas and some solid areas. The immunohistochemical analysis showed cells positive for epithelial membrane antigen (EMA), lying in a myxoid stroma. Proliferation index evaluated with Ki-67 was 2%. The surgical margins were negative. These features have allowed for the diagnosis of myxopapillary ependymoma grade 2 (WHO 2021 classification), radically excised. Coccygectomy was deemed unnecessary. Following histological diagnosis, given the metastatic potential of the lesion, staging with total body CT and CNS MRI was performed, which revealed no other foci of disease. At one-year follow-up (Figure 1B), performed with quarterly evaluations with lumbosacral MRI, the boy is in complete remission.

## 3. Discussion

Ependymoma represents the third most frequent CNS tumor in childhood and is mainly localized in the posterior cranial fossa where spinal localization is more frequent among adults [7,8]. The brain tumor most frequently originates or metastasizes outside the CNS [3,4,5]. More than a century ago, Mallory et al. first described the case of an ependymoma that developed in the subcutaneous tissue of the sacrococcygeal region [9]. Since then, only a few cases have been reported in literature with less than 50 reported cases occurring in childhood during the last 35 years (Table 1) [4,10,11,12,13,14,15,16,17,18,19,20,21,22,23,24,25,26,27,28,29,30,31,32,33,34,35,36]. They occur more frequently in the sacrococcygeal region, followed by the pelvic region, while cases of onset in the liver, lung, or mediastinum are rarer and mainly described in adulthood [3,5]. As in the cases we have described, the most frequent histological type among sacrococcygeal ependymomas, reflecting their spinal counterpart, is the myxopapillary (grade 2 WHO), which is characterized by tumor cells arranged in a papillary manner around vascularized myxoid stromal cores, strongly and diffusely positive for GFAP and S-100 on immunohistochemical analysis [37]. Conversely, classical ependymomas become more frequent in the pelvic or mediastinal region, while more aggressive histological subtypes have been described less frequently, sometimes as areas of undifferentiation associated with a myxopapillary component [27,29]. Recently, the first case of giant cell ependymoma of the sacrococcygeal region occurring in childhood was reported by Planas and colleagues [36].

**Figure 2:**
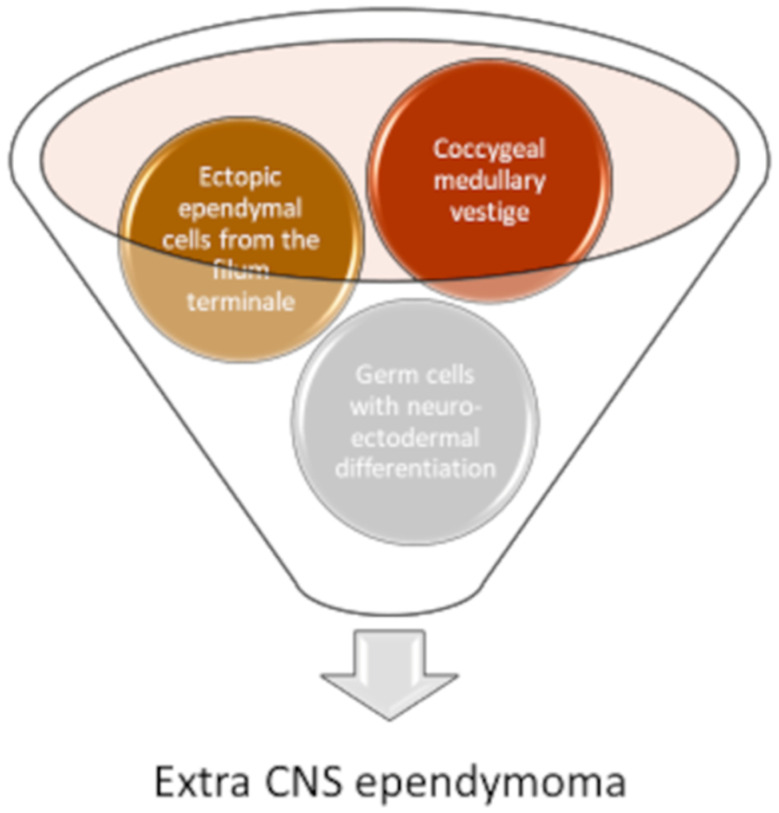
There are three hypotheses regarding the origin of the extra CNS ependymomas: From the coccygeal medullary vestige (an ependymal lined cavity forming from the remnants of the caudal portion of neural tube); from ectopic ependymal cells originating from the filum terminale; from primordial germ cells with neuroectodermal differentiation.

While it is universally accepted that CNS ependymomas develop from the ependymal cells lining in the ventricles and the central canal, the pathogenesis of extraspinal ependymomas is still debated, with three main hypotheses reported in the literature [3,4,6] (Figure 2). The first hypothesis suggests that they arise from the coccygeal medullary vestige, that is an ependymal lined cavity forming from the remnants of the caudal portion of neural tube: ependymal rests have often been found in random autopsies [38,39]. Furthermore, this hypothesis could explain the high prevalence of the myxopapillary subtype, which typically originates from the filum terminale. Another hypothesis is that these tumors develop from ectopic ependymal cells originating from the filum terminale, supported by the correlation between neural arch defects and the onset of extraspinal ependymomas [40,41,42,43]. Other authors suggest that they may originate from primordial germ cells with neuroectodermal differentiation: the latter hypothesis could explain the adnexal and mediastinal localizations, and the positivity for estrogen and progestin receptors often found in these neoplasms [3].

The clinical presentation varies according to the onset region: in their review of sacrococcygeal ependymomas, Lien and colleagues found an asymptomatic mass as the most frequent initial presentation (about 50% of cases), while rarer symptoms such as pain, tenesmus, constipation, or signs of neuropathy were encountered [5]. The cases we have described also presented with a mass without associated symptoms, and the initial presumptive diagnosis were epidermoid cyst and germ cell tumor, respectively. In fact, given their localization and the propensity to drain which could suggest a diagnosis of pilonidal cyst or other benign tumors, misdiagnosis at onset is very common, occurring in virtually all cases described in the literature [5,28,34,44]. Thus, with any soft tissue lesion of the sacrococcygeal region, the differential diagnosis must include also ependymoma, in addition to sacrococcygeal teratoma, neurogenic tumor, soft tissue sarcoma, and metastatic carcinoma. [6] Pelvic ependymomas are usually more extensive at diagnosis and may be associated with bowel or bladder dysfunction, or more rarely, with signs or symptoms of neuropathy, such as saddle anesthesia [4,45,46]. Despite the greater prevalence of low-grade histological subtypes, extra CNS ependymomas are more frequently metastatic at onset than their intra-axial counterpart: approximately 15% of pediatric cases reported in literature in the past 35 years have metastases at onset, mostly localized to the inguinal lymph nodes [4,5,47]. This apparent discrepancy can be explained by easier access to blood and lymphatic vessels due to the absence of the blood brain barrier [4]. Extra CNS ependymomas arising in childhood have a tendency for both local and metastatic recurrences, the inguinal nodes being the most common localization. Relapse occurs in about 20% of cases described in the last 35 years, even more than 10 years after the initial remission [4,10,11,12,13,14,15,16,17,18,19,20,21,22,23,24,25,26,27,28,29,30,31,32,33,34,35,36]. Despite this, the prognosis is good: in the past 35 years, only one death in 39 cases has been reported in the literature [17].

Currently, there are no standardized guidelines for the management of extra CNS ependymomas. The most effective treatment seems to be complete surgical resection: Sonneland et al. in their study on 77 cases found a lower relapse rate in patients undergoing en bloc resection than in those undergoing piecemeal resection (10% vs 19%). Gross total resection is associated with better OS compared to subtotal resection (19 years vs. 14 years) [45]. Coccygectomy is recommended if the tumor adheres to the bone [21,48]. It could also play a role in the prevention of local recurrence, as suggested by Aktug and colleagues: in their review of 22 pediatric sacrococcygeal ependymomas, they found no recurrence in patients undergoing coccygectomy, while 5 of 7 patients who had no coccyx removed experienced a local recurrence [23]. On the other hand, cases of relapse despite coccygectomy are reported in the literature [4,29]. In our review, we found four recurrences (two local and two metastatic) out of 17 patients who did not undergo coccygectomy. Furthermore, relapse occurred in two of the 11 patients in which the coccyx was removed; among the latter, both were metastatic with no evidence of local recurrence. These data regarding coccygectomy, which seem to suggest a role only in preventing local recurrence, do not currently allow to recommend it in all cases, but need to be verified in a larger populations in order to be able to be considered significant. In this respect, removal of coccyx en bloc with specimen essential for local control in management of sacrococcigeal malignant germ cell tumors. If ruptured, invades surrounding structures, or unresectable, initial biopsy and neoadjuvant chemotherapy should be given. It could be speculated that CNS ependymomas may require similar approach/management, but further studies are needed to validate this option and evaluate the impact on outcome [49,50].

The role of radiotherapy in the treatment of pediatric extra CNS ependymomas is less clear than that of surgery. Unlike its demonstrated role in the treatment of the intracranial disease, there is no clear evidence regarding its real effectiveness in the extra CNS setting. However, there is agreement in the literature to recommend it in the case of subtotal resection or inoperable mass, after the removal of recurrent lesions and in the case of metastatic disease [4,5,21,48]. Most authors recommend a total dose of 50 Gy [40,48], but there are also cases where a lower dose (30–45 Gy) proved to be sufficient [51].

The role of chemotherapy in treating children affected by intracranial ependymomas remains unproven despite intensive investigation [52,53]. Thus, it is not surprising that its use in the management of the extra CNS counterpart is still a matter of debate. Several drugs have been used, with conflicting results. Rao et al. used cisplatin, etoposide and bleomycin in both a neoadjuvant and adjuvant setting, achieving complete remission [24]. Schiavello et al. Administered, to a metastatic patient, neoadjuvant chemotherapy consisting of vincristine, carboplatin, epirubicin, actinomycin-D, ifosfamide and etoposide, achieving a partial response, and followed by radiotherapy and surgical resection. In the same paper, another patient with lymph node recurrence was treated with adenectomy followed by adjuvant chemotherapy (cisplatin, etoposide, doxorubicin, vincristine and cyclophosphamide), resulting in complete remission. They also reported the use of high-dose chemotherapy (melphalan) followed by autologous stem cell transplantation in a patient with both local and metastatic recurrence; the patient finally underwent radiotherapy on primitive lesion and is alive 15 years since follow-up [4]. The use of target therapy as tyrosine kinase inhibitors or hormonal suppressive agents has been reported in adult patients, with moderate results [3,54,55]. 

Despite the generally good prognosis of this disease, a long-term follow-up must be warranted, given the aforementioned possibility of relapse even after many years.

## 4. Conclusions

Extra-CNS ependymoma is exceedingly rare and difficult to diagnose by imaging. The rate of metastastic disease is not trivial and warrants total body stadiation in all cases. The mainstay of treatment is radical surgery, including resection of coccygeal bone when involved by infiltrative margins. The role of adjuvant treatment is limited to selected cases. Collaborative studies are needed to better define the optimal management of these patients.

## Figures and Tables

**Table 1:** Extra SNC ependymomas occurring in children during the last 35 years.

Authors	Age (Years)	Sex	Localization	Histology	Metastases	First-Line Treatment	Coccygectomy	Recurrence	Follow-up
Ciraldo 1986 [10]	0.75	F	Subcutaneous sacrococcygeal	Myxopapillary	No	Surgery	Yes	No	Alive
Chou 1987 [11]	16	F	Subcutaneous sacrococcygeal	Myxopapillary	NA	Surgery	No	NA	NA
Kramer 1988 [12]	15	M	Subcutaneous sacrococcygeal	Myxopapillary	No	Surgery	No	Yes, local and metastatic (inguinal nodes)	20 years
Marc’Hadour 1991 [13]	14	F	Subcutaneous sacrococcygeal	Myxopapillary	No	Surgery	No	No	2 years
Gupta 1992 [14]	1.5	M	Subcutaneous sacrococcygeal	Myxopapillary	Inguinal node	Surgery	No	Yes, local	NA
Serour 1993 [15]	8	M	Subcutaneous sacrococcygeal	Myxopapillary	No	Surgery	Yes	No	20 months
Botti 1994 [16]	10	NA	Subcutaneous sacrococcygeal	Myxopapillary	Left buttock	Surgery	No	No	NA
Kline 1996 [17]	0.67	F	Subcutaneous sacrococcygeal	Myxopapillary	Inguinal nodes	Surgery	No	Yes, metastatic (inguinal nodes, peritoneum)	Death
Webber 1996 [18]	newborn	M	Presacral	NA	No	Surgery	NA	Yes, local	NA
Sawyer 1998 [19]	13	F	Subcutaneous sacrococcygeal	Myxopapillary	No	Surgery	No	NA	NA
Ilhan 1998 [20]	8	M	Subcutaneous sacrococcygeal	Myxopapillary	No	Surgery	Yes	No	20 months
Johnson 1999 [21]	7	M	Subcutaneous sacrococcygeal	Grade II	No	Surgery	Yes	No	8 years
Grubnic 1999 [22]	8	M	Subcutaneous sacrococcygeal	Myxopapillary	NA	Surgery	Yes	No	NA
Aktuǧ 2000 [23]	5	M	Subcutaneous sacrococcygeal	Myxopapillary	No	Surgery	Yes	No	3 years
Rao 2002 [24]	1.33	F	Presacral	Myxopapillary	No	Surgery + CT	Yes	No	Alive
Akpolat 2003 [25]	7	M	Subcutaneous sacrococcygeal	Myxopapillary	No	Surgery	No	No	Alive
Tröbs 2006 [26]	9	M	Subcutaneous sacrococcygeal	Myxopapillary	No	Surgery	Yes	No	NA
Beschorner 2007 [27]	1.2	M	Subcutaneous sacrococcygeal	Grade IV (Myxopapillary + areas of ependymoblastic differentiation)	No	Surgery	No	No	Alive
Alexiou 2012 [28]	13	F	Subcutaneous sacrococcygeal	Myxopapillary	No	Surgery + RT	No	No	8 months
Chakraborti 2012 [29]	1	F	Subcutaneous sacrococcygeal	Grade IV (Myxopapillary + areas of anaplastic differentiation)	No	Surgery	Yes	Yes, metastatic (inguinal nodes)	Alive
Cimino 2014 (7 patients) [30]	0–17 (range), 7.4 (mean)	4F/3M	NA	Myxopapillary	NA	NA	NA	No	NA
Dogan 2016 [31]	9	F	Subcutaneous sacrococcygeal	Myxopapillary	No	Surgery	No	No	Alive
Amin 2018 [32]	8	F	Subcutaneous sacrococcygeal	Myxopapillary	NA	Surgery	Yes	No	Alive
Schiavello 2018 [4]	14	M	Presacral	Grade II	No	Surgery	No	No	6 years
	8	F	Presacral	Grade II	inguinal nodes, osseus	CT + RT + Surgery	No	No	26 years
	4	F	Subcutaneous sacrococcygeal	Myxopapillary	Parauterine nodes	Surgery	Yes	Yes, metastatic (neoplastic iliac thrombosis, lung, broad ligament, retroperitoneal and mediastinal nodes)	15 years
	12	M	Subcutaneous sacrococcygeal	Grade II	No	Surgery	No	No	11 years
	16	M	Subcutaneous sacrococcygeal	Myxopapillary	No	Surgery	No	No	11 years
	7	F	Subcutaneous sacrococcygeal	Myxopapillary	No	Surgery	No	Yes, metastatic (inguinal nodes)	33 years
Rogers 2018 [33]	12	F	Subcutaneous sacrococcygeal	Myxopapillary	NA	Surgery	No	No	1 year
Gupta 2020 [34]	9	M	Subcutaneous sacrococcygeal	Myxopapillary	No	Surgery	No	No	18 months
Thejeel 2020 [35]	16	F	Subcutaneous sacrococcygeal	Myxopapillary	No	Surgery	NA	NA	NA
Planas 2021 [36]	12	F	Subcutaneous sacrococcygeal	Giant cell	No	Surgery	No	No	2 years

## Data Availability

Not applicable.
